# Inhibitory Activity of (+)-Usnic Acid against Non-Small Cell Lung Cancer Cell Motility

**DOI:** 10.1371/journal.pone.0146575

**Published:** 2016-01-11

**Authors:** Yi Yang, Thanh Thi Nguyen, Min-Hye Jeong, Florin Crişan, Young Hyun Yu, Hyung-Ho Ha, Kyung Hee Choi, Hye Gwang Jeong, Tae Cheon Jeong, Kwang Youl Lee, Kyung Keun Kim, Jae-Seoun Hur, Hangun Kim

**Affiliations:** 1 Korean Lichen Research Institute, Sunchon National University, Sunchon, Republic of Korea; 2 Faculty of Natural Science and Technology, Tay Nguyen University, Buon Ma Thuot, Vietnam; 3 Department of Taxonomy and Ecology, Faculty of Biology and Geology, Babeș-Bolyai University, Cluj-Napoca, Romania; 4 College of Pharmacy and Research Institute of Life and Pharmaceutical Sciences, Sunchon National University, Sunchon, Republic of Korea; 5 College of Pharmacy, Chungnam National University, Daejeon, Republic of Korea; 6 College of Pharmacy, Yeungnam University, Gyeongsan, Korea; 7 College of Pharmacy, Chonnam National University, Gwangju, Korea; 8 Medical Research Center for Gene Regulation, Chonnam National University Medical School, Gwangju, Republic of Korea; Aix-Marseille University, FRANCE

## Abstract

Lichens are symbiotic organisms that produce various unique chemicals that can be used for pharmaceutical purposes. With the aim of screening new anti-cancer agents that inhibit cancer cell motility, we tested the inhibitory activity of seven lichen species collected from the Romanian Carpathian Mountains against migration and invasion of human lung cancer cells and further investigated the molecular mechanisms underlying their anti-metastatic activity. Among them, *Alectoria samentosa*, *Flavocetraria nivalis*, *Alectoria ochroleuca*, and *Usnea florida* showed significant inhibitory activity against motility of human lung cancer cells. HPLC results showed that usnic acid is the main compound in these lichens, and (+)-usnic acid showed similar inhibitory activity that crude extract have. Mechanistically, β-catenin-mediated TOPFLASH activity and KITENIN-mediated AP-1 activity were decreased by (+)-usnic acid treatment in a dose-dependent manner. The quantitative real-time PCR data showed that (+)-usnic acid decreased the mRNA level of CD44, Cyclin D1 and c-myc, which are the downstream target genes of both β-catenin/LEF and c-jun/AP-1. Also, Rac1 and RhoA activities were decreased by treatment with (+)-usnic acid. Interestingly, higher inhibitory activity for cell invasion was observed when cells were treated with (+)-usnic acid and cetuximab. These results implied that (+)-usnic acid might have potential activity in inhibition of cancer cell metastasis, and (+)-usnic acid could be used for anti-cancer therapy with a distinct mechanisms of action.

## Introduction

Lung cancer is the leading cause of cancer death in developed countries. Due to the lack of efficient treatment for advanced disease, the prognosis of lung cancer is still poor, with less than 15% surviving 5 years after diagnosis [1]. Adjacent invasion and distant metastasis are the major causes of cancer-related death [2]. Therefore a search for inhibitors for cancer cell invasion and migration ability could reveal a new therapy for cancer treatment. Although herbs have been employed in the treatment of cancers for thousands of years, they remain a very important source of biologically active products. The aim of this study was to identify potential therapeutic agents to improve the survival of patients with lung cancer metastasis.

Lichens are symbiotic organisms that produce a large number of bioactive substances over 800 [3], comprising many classes of compounds: amino acid derivatives, sugar alcohols, aliphatic acids, γ-, δ- and macrocyclic lactones, monocyclic aromatic compounds, quinones, chromones, xanthones, dibenzofuranes, depsides, depsidones, depsones, terpenoids, steroids, carotenoids [4] and diphenyl ethers [5, 6]. Slowly growing organisms in low-resource habitats produce higher levels of defense chemicals [7]. Therefore, lichens are a source of unique chemical agents of which some have already been proved to be effective against various cancer *in vitro* models [8]. Here, the current study examined the inhibitory activity of seven lichen species collected from the Romanian Carpathian Mountains against migration and invasion ability of human lung cancer cells and further investigated the possible molecular mechanisms underlying their anti-metastatic activity to identify potential compounds for novel anti-metastasis agents.

## Material and Methods

### Preparation of lichen extracts

Lichen specimens used in this study, collected from Romania in 2011, were identified at the Korean Lichen Research Institute (KoLRI), Sunchon National University, Korea. Briefly, thalli of lichen were collected from Romania in 2011 during the field trip in the National Park Călimani (47°07'28.6"N, 25°13'34.8"E) and the Natural Park Bucegi (45°20'21.7"N, 25°27'41.4"E) organized by Dr. Crişan at Babeş-Bolyai University, Cluj-Napoca, Romania [9]. The permit to collect lichen specimens from those locations was issued by the Administration of the National Park Călimani and the Administration of the Natural Park Bucegi, with the approval of the Commission for Protection of Natural Monuments (Romanian Academy). The field studies did not involve any endangered or protected species. The duplicates were deposited into the Korean Lichen and Allied Bioresource Center (KOLABIC) in the Korean Lichen Research Institute (KoLRI), Sunchon National University, Korea. The dried thalli of the lichens were extracted with acetone at room temperature for 48 h. The acetone extracts were then filtered and dried in rotary vacuum evaporator at 45°C. The dry extracts were dissolved in dimethylsulfoxide (DMSO) as 5 mg/ml concentration (1000×) for all experiments. Seven Romanian lichen species and their voucher specimen numbers used in this study were listed in Table 1.

**Table 1:** Seven Romanian lichen species used in this study.

Collection No.	Family	Lichen species	Known lichen substances	Reference
RO11025	*Parmeliaceae*	*Alectoria samentosa*	(-)-Usnic acid, Physodic acid, 8’-O-ethyl-P-alectoronic acid, Alectosarmentin	[13]
RO11045	*Parmeliaceae*	*Flavocetraria nivalis*	(±)- Usnic acid, Isousnic acid, Divaricatic acid, *p*-Hydroxybenzoic acid, Vanillic acid	[14–17]
RO11084	*Parmeliaceae*	*Alectoria ochroleuca*	Diffractaic acid, (-)-Usnic acid, Isousnic acid, Friedelin, Barbatic acid	[16, 18]
RO11111	*Parmeliaceae*	*Bryoria capillaris*	Alectorialic acid, Barbatolic acid	[19]
RO11166	*Parmeliaceae*	*Hypogymnia physodes*	Atronorin, Physodalic acid, Protocetraric acid, Physodic acid	[18, 19]
RO11176	*Parmeliaceae*	*Usnea florida*	(+)-Usnic acid, Barbatic acid, Salazinic acid, Norstic acid, β-Orcinol depsidones, (±)-Thamnolic acid, Stictic acid	[18, 20]
RO11209	*Parmeliaceae*	*Evernia divaricata*	Divaricatic acid	[19]

### High performance liquid chromatography (HPLC) analysis of lichen material

Acetone extract of lichen thalli at a concentration of 5 mg/ml were subjected to high performance liquid chromatography (HPLC) analyses (LC-20A; Shimadzu, Kyoto, Japan) on a YMC-Pack ODS-A (150 × 3.9 mm I.D.) reversed-phase column containing fully end-capped C18 material (particle size, 5 μm; pore size, 12 nm). Elution was performed at a flow rate of 1 ml/min under the following conditions before subsequent injection: column temperature, 40°C; solvent system, methanol: water: phosphoric acid (80: 20: 1, v/v/v). Analyses were monitored by a photodiode array detector (SPD-M20A; Shimadzu) with a range of 190~800 nm throughout the HPLC run. Observed peaks were scanned between 190 and 400 nm. The standard used for salazinic acid (t_R_ = 2.27 ± 0.2 min) was isolated from lichen *Lobaria pulmonaria*. Usnic acid used in our study was purchased from Sigma-Aldrich (St. Louis, USA) (329967-5G). Voucher specimens were deposited in the herbarium of the Lichen & Allied Bioresource Centre at the Korean Lichen Research Institute, Sunchon National University, South Korea.

### Liquid chromatography-mass spectrometry (LC-MS) and optical rotation analaysis of lichen material

LC—MS spectra were recorded on a spectrometer with an electrospray ionization source using Agilent 6460 triple Quadrupole LC/MS. The values of optical rotation were measured at 25°C using Jasco P-1010 polarimeter with a sodium lamp, and described as follows: [α]D, T (c (g/100 mL), solvent). Specific rotation of pure (+)-usnic acid (Sigma-Aldrich, St. Louis, USA) is a physical property of at a given wavelength and temperature and can be looked up in literature.

### Cell culture

The human lung cancer cells including A549, H460, H1650, and H1975 were cultured in RPMI 1640 culture medium supplemented with 10% fetal bovine serum, 1% Penicillin-Streptomycin solution under a humidified 5% CO_2_ atmosphere at 37°C.

### Wound healing assay

A549 cells were plated at a density of 2.5 × 10^5^ cells/well on 6-well tissue culture plates (Corning, New York, USA) and grown overnight to confluence. Monolayer cells were scratched with a pipette tip to create a wound. The cells were then washed twice with serum-free RPMI 1640 to remove floating cells and incubated in RPMI1640 culture medium supplemented with 2% FBS with 5 μg/ml of the lichen extract or 5 μM usnic acid. Photographs of cells were taken at 0, 24, 48, and 72 h after wounding to measure the width of the wound. For each sample, an average of five wound assays was taken to determine the average rate of migration at a given concentration of acetone extract or usnic acid. Experiments were repeated at least three times.

### Invasion assay

Invasion assays were performed in transwell chambers (Corning, New York, USA) with 8μm pore size polycarbonate membrane coated with 1% gelatin. Cells were plated at 2 × 10^5^ cells/well in RPMI1640 containing 0.2% bovine serum albumin in the upper compartment of the chamber with or without 5 μg/ml crude lichen extracts. Then RPMI1640 medium with 10 μg/ml fibronectin was added to the lower chamber to serve as a chemotactic agent. After 48-h incubation, the cells in the upper chamber were fixed with Diff Quik kit (Sysmex, Kobe, Japan). Then the cells inside the chamber were mechanically removed from the membrane with a cotton swab, and the cells adhering to the under-side of the membrane were stained and counted under light microscope (5 fields per chamber). Each invasion assay was repeated in three independent experiments. The results are expressed as the mean number of cells migrating per high-power field.

### Reporter assay

HEK293T cells were plated into 24-well plates 12 h before transfection. After transfection of the TOPFLASH or AP-1 reporter plasmid with the respective activator, β-catenin or KITENIN, cells were treated with usnic acid for 48 h and then analyzed using a Dual-Luciferase^®^ reporter assay system (Promega, Madison, WI, USA). The Renilla luciferase reporter plasmid (pRL-TK) was used as the internal control for the transfection efficiency. The experiments were performed in triplicate, and at least three results from independent experiments were included in the analysis. Fold changes were calculated using values normalized to Renilla luciferase activity.

### Quantitative real-time PCR

The quantitative real-time PCR was performed as described previously [9]. Briefly, total RNA was isolated from human lung cancer cells by using RNAiso Plus (TaKaRa, Otsu, Shiga 520–2193, Japan) according to the manufacturer’s instructions. Total RNA (1 μg) from each group of treated cells was converted to cDNA using a M-MLV reverse Transcriptase kit (Invitrogen, Carlsbad, USA) and SYBR green (Enzynomics, Seoul, Korea). The primers used for real-time PCR were Cyclin D1 (forward) 5’-ccgtccatgcggaagatc-3’ and (reverse) 5’-gaagacctcctcctcgcact-3’; c-myc (forward) 5’-aatgaaaaggcccccaaggtagttatcc-3’ and (reverse) 5’-gtcgtttccgcaacaagtcctcttc-3’; CD44 (forward) 5’-tgccgctttgcaggtgtat-3’ and (reverse) 5’-ggcctccgtccgagaga-3’; GAPDH (forward) 5’-atcaccatcttccaggagcga-3’ and (reverse) 5’-agttgtcatggatgaccttggc-3’. Real-time PCR reaction and analysis were performed using CFX (Bio-Rad, Hercules, USA).

### Affinity Precipitation of Cellular GTPases

The cellular Rac1 and Cdc42 activities were determined using GST-RBD/PBD as previously described [10, 11]. Briefly, the cells were lysed in lysis buffer (50 mM Tris, pH 7.4, 1% Triton X-100, 0.5% sodium deoxycholate, 0.1% SDS, 500 mM NaCl, 10 mM MgCl_2_, and protease inhibitor mixture). The lysates were incubated with GST- RBD/PBD beads at RT for 1 h. The beads were then washed four times with washing buffer (50 mM Tris, pH 7.4, 1% Triton X-100, 150 mM NaCl, 10 mM MgCl_2_, and protease inhibitor mixture). The bound Rac1 and Cdc42 proteins were detected by immunoblotting using a monoclonal antibody against Rac1 (MILLIPORE 05–389) and Cdc42 (SANTA CRUZ SC-87). The relative activity of each GTPase was determined by quantifying each band of GTP-bound GTPase and the total amount of GTPase using Multi-Gauge 3.0, and the values of the GTP-bound bands were normalized to the value of the total amount. All results were determined using three different exposures from at least three independent experiments.

## Results

### Inhibition of A549 cell motility by lichen extracts

**Fig 1:**
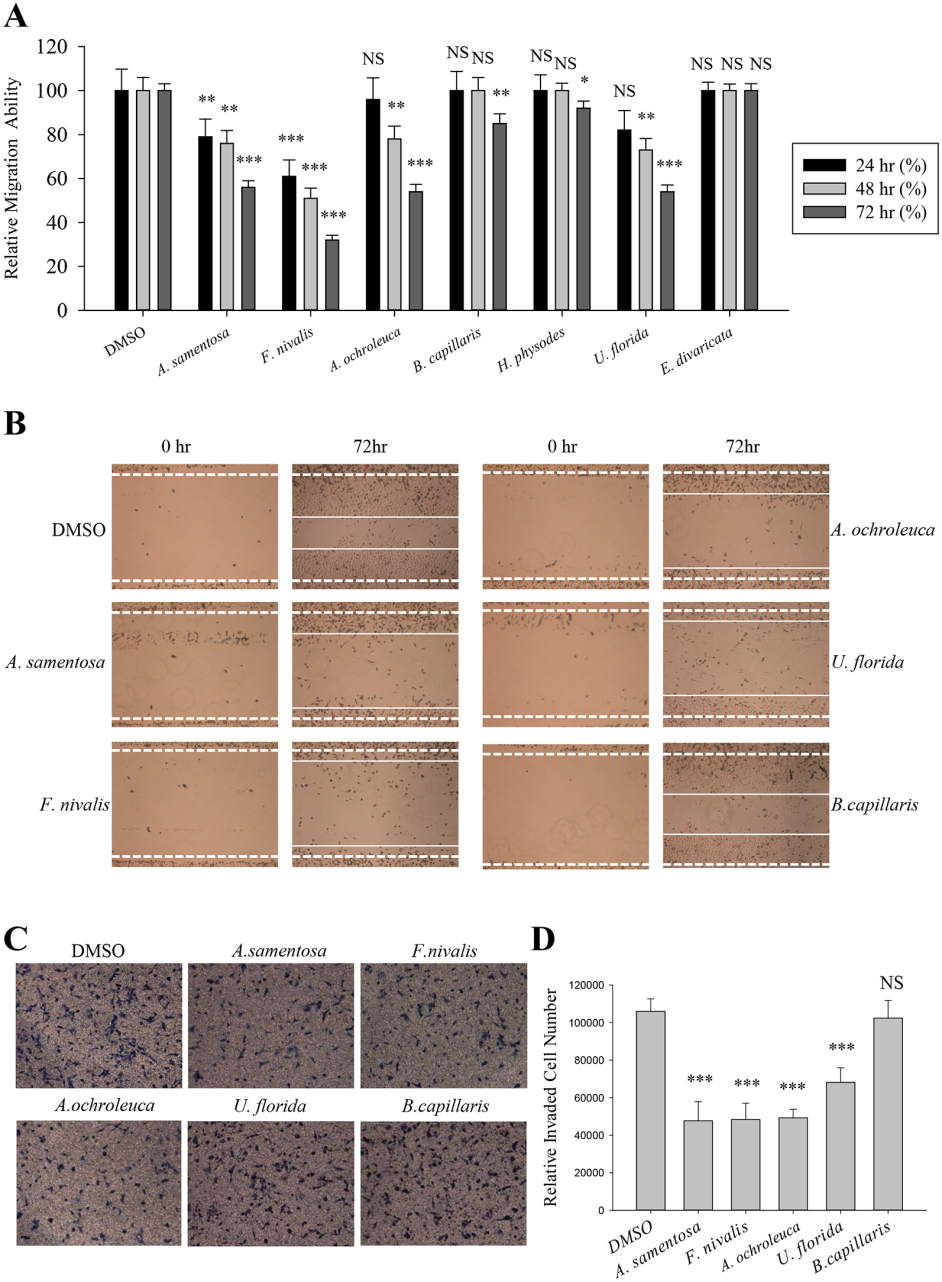
Inhibition of A549 cell motility by acetone extracts of lichens. (A–B) Quantitative analysis of migration assay of A549 cells treated with 5 μg/ml of acetone extracts of *Alectoria samentosa*, *Flavocetraria nivalis*, *Alectoria ochroleuca*, *Bryoria capillaris*, *Hypogymnia physodes*, *Usnea florida* and *Evernia divaricata* (A), and representative images of migration assay of A549 cells treated with the extracts of *A*. *samentosa*, *F*. *nivalis*, *A*. *ochroleuca*, *U*. *florida* and *B*. *capillaris* (B). (C-D) Invasion assay of A549 cells treated with 5 μg/ml of acetone extracts of *A*. *samentosa*, *F*. *nivalis*, *A*. *ochroleuca*, *U*. *florida* and *B*. *capillaris* (C), and quantitative analysis of invaded cell numbers in each group (D). Representative images were shown from three independent experiments, n = 3. Data represent mean ± S.E.M. (standard error of the mean). ***p<0.001; NS, no significant difference compared to 0.01% DMSO-treated A549 cells.

Cell migration plays a crucial role during cancer metastasis. To find the anti-migratory lichen secondary metabolite on human lung cancer cells, wound healing assay was performed among seven acetone extracts of Romanian lichens listed in Table 1. Lichens produce unique polyketide secondary metabolites including depsides, depsidones, dibenzofurans, and depsones; and these hydrophobic compounds were normally extracted with acetone [12]. As shown in Fig 1A, *Alectoria samentosa*, *Flavocetraria nivalis*, *Alectoria ochroleuca*, and *Usnea florida* inhibited A549 cell migration at a concentration of 5 μg/ml. The length between the edges of the wound at 72 h with these candidates were significantly wider than that in the DMSO-treated group or the non-active sample (*Bryoria capillaris*). In particular, *F*. *nivalis* showed more than 60% inhibitory activity compared to control (Fig 1A and 1B).

The effects of acetone extracts of *A*. *samentosa*, *F*. *nivalis*, *A*. *ochroleuca*, and *U*. *florida* on A549 cell invasion were then determined using transwell chamber invasion assay. As a result, the lichen extracts significantly decreased the invaded cell numbers by as much as 50% compared with DMSO or *B*. *capillaries* (negative control) (Fig 1C and 1D). These findings demonstrated that acetone extracts of *A*. *samentosa*, *F*. *nivalis*, *A*. *ochroleuca*, and *U*. *florida* inhibited both migration and invasion ability of A549 lung cancer cells.

### Usnic acid is the active inhibitor from the lichen species

**Fig 2:**
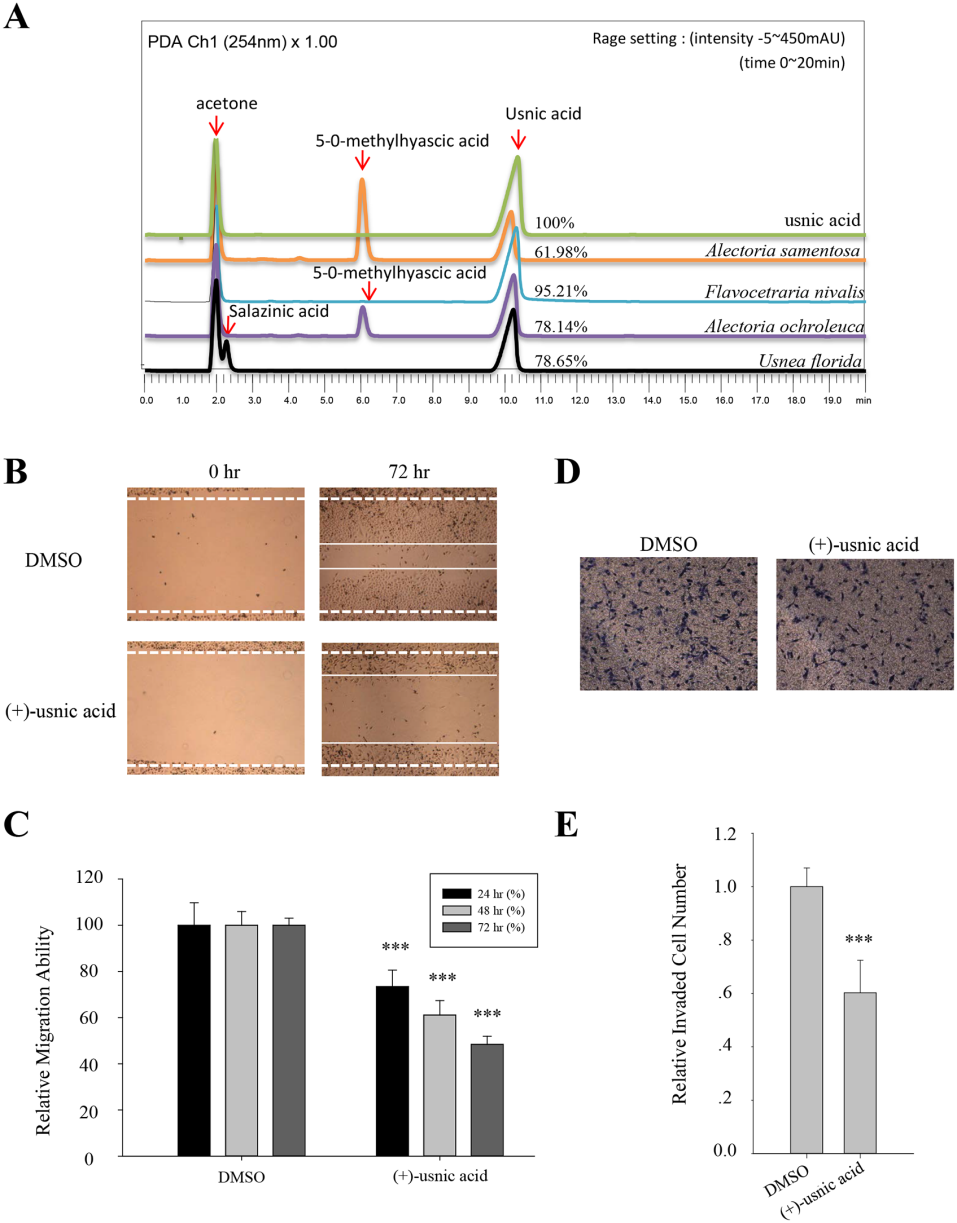
Identification of lichen secondary metabolite from candidate lichens. (A) High performance liquid chromatography (HPLC) analysis of lichen acetone extracts. The %intensity of peak for the usnic acid in the extract at a concentration of 5 mg/ml was obtained by comparing to that of peak for pure 5 mg/ml usnic acid. (B–C) Migration assay of A549 cells treated with 5 μM of (+)-usnic acid (B), and quantitative analysis of wound length (C). (D–E) Invasion assay of A549 cells treated with 5 μM of (+)-usnic acid (D), and quantitative analysis of invaded cell numbers in each group (E). Representative images are shown from three independent experiments, n = 3. Data represent mean ± S.E.M. (standard error of the mean). ***p<0.001; NS, no significant difference compared to 0.01% DMSO-treated A549 cells.

**Fig 3:**
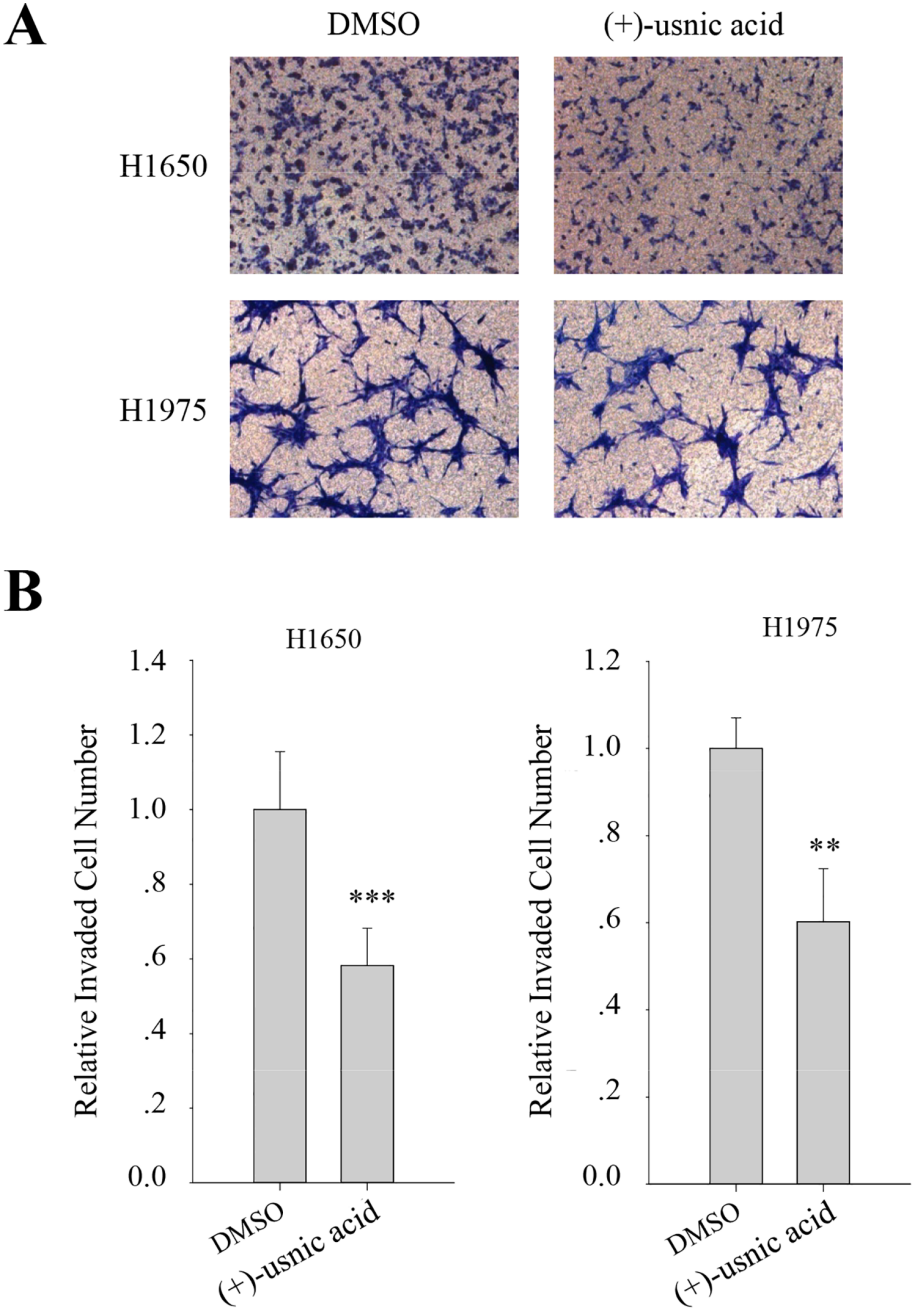
(+)-Usnic acid inhibits invasion of H1650 and H1975 human lung cancer cell. (A-B) Invasion assay of H1650, and H1975 cells treated with 5 μM of (+)-usnic acid (A), and quantitative analysis of invaded cell numbers in each cell line (B). Representative images are shown from three independent experiments, n = 3. Data represent mean ± S.E.M. (standard error of the mean). **p<0.01; ***p<0.001; NS, no significant difference compared to 0.01% DMSO-treated A549 cells.

To identify the components of the acetone extract of lichen, *A*. *samentosa*, *F*. *nivalis*, *A*. *ochroleuca*, and *U*. *florida* extracts were run on HPLC. As shown in Fig 2A, usnic acid was identified as the main compound in all four of these candidates after comparison with the internal standard of purified (+)-usnic acid (Sigma-Aldrich, St. Louis, USA), and these were consistent with previous report (Table 1) [13–20]. Identity of usnic acid and their optical status were analyzed by LC-MS analysis and optical activity analysis, respectively (S1 File). The %intensity of peak for the usnic acid in the candidate lichens at a concentration of 5 mg/ml was obtained by comparing to that of peak for pure 5 mg/ml usnic acid. It is worth noting that usnic acid content is highest in *F*. *nivalis* extract, which may explain its potent inhibitory effect on cell migration (Fig 2A). It was speculated that (-)-usnic acid has similar or more potent inhibitory activity on cell motility as acetone extract of *A*. *samentosa* and *A*. *ochroleuca* which are known to have (-)-usnic acid as their subcomponent [13, 16, 18] showed similar or more potent inhibitory activity on migration and invasion, respectively, than (+)-usnic acid containing *U*. *florida* (Fig 1). In our previous report, we showed that acetone extract of lichen *F*. *cucullata* and its component, usnic acid, inhibited tumorigenicity and motility of cancer cells [9]. In accordance with this, (+)-usnic acid at concentration of 5 μM significantly inhibited the migration and invasion of A549 cells (Fig 2). At this concentration, usnic acid did not show cytotoxicity and/or inhibit cell proliferation (IC_50_ value of usnic acid on A549 cells = 65.3 ± 0.65 μM) [9]. As shown in Fig 2B–2E, inhibitory activity of (+)-usnic acid at 5 μM is as high as 50% at 72 h treatment for migration and is around 40% at 48 h treatment for invasion. To further examine the inhibitory activity of (+)-usnic acid on the other lung cancer cells, invasion assay was performed using H1650, and H1975 cells. As a result, (+)-usnic acid treatment significantly decreased invaded cell number of H1650 and H1975 cells at a concentration of 5 μM (Fig 3A). The quantitative analysis revealed that inhibition was as high as 40% in both cells compared with vehicle-treated cells (Fig 3B). Together, the results demonstrated that (+)-usnic acid has inhibitory activity against cell motility of human lung cancer cells.

### (+)-Usnic acid decreases β-catenin-mediated TOPFLASH activity and KITENIN-mediated AP-1 activity

**Fig 4:**
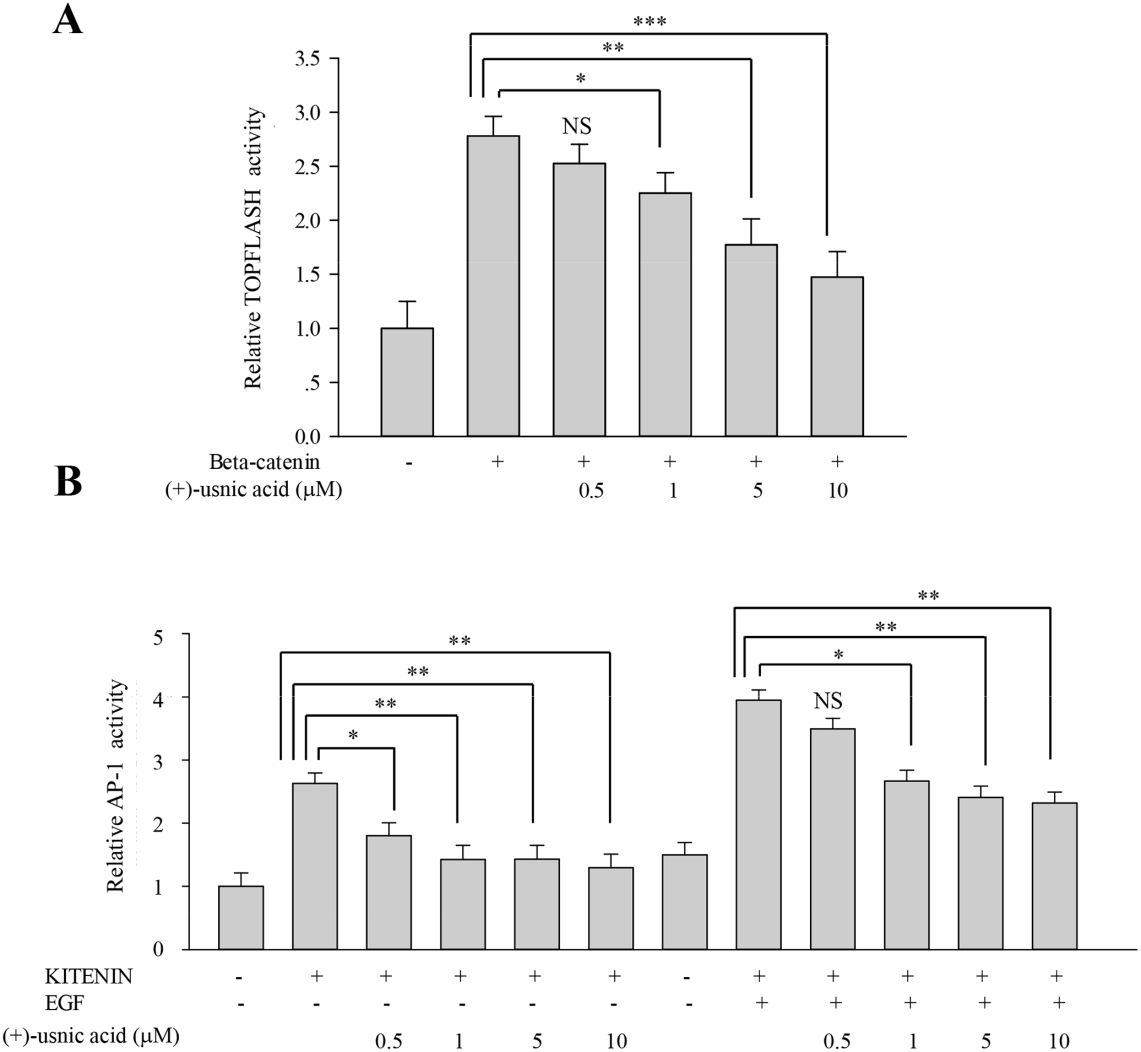
(+)-Usnic acid decreases β-catenin-mediated TOPFLASH activity and KITENIN-mediated AP-1 activity. (A) β-Catenin-mediated transcriptional activity of TOPFLASH promoter was decreased by (+)-usnic acid treatment. HEK 293T cells were transfected with β-catenin and TOPFLASH reporter plasmid. After 12 h transfection, cells were treated with (+)-usnic acid for 48h. (B) KITENIN-mediated transcriptional activity of AP1 promoter was decreased by (+)-usnic acid treatment. The HEK 293T cells were transfected with KITENIN and AP-1 reporter plasmid. After 12 h transfection, cells were treated with (+)-usnic acid for 48h with or without EGF. Experiments were performed in at least three independent cultures, n = 3. Data represent mean ± S.E.M. (standard error of the mean). *p<0.05; **p<0.01; ***p<0.001; NS, no significant difference compared to 0.01% DMSO-treated HEK 293T cells.

**Fig 5:**
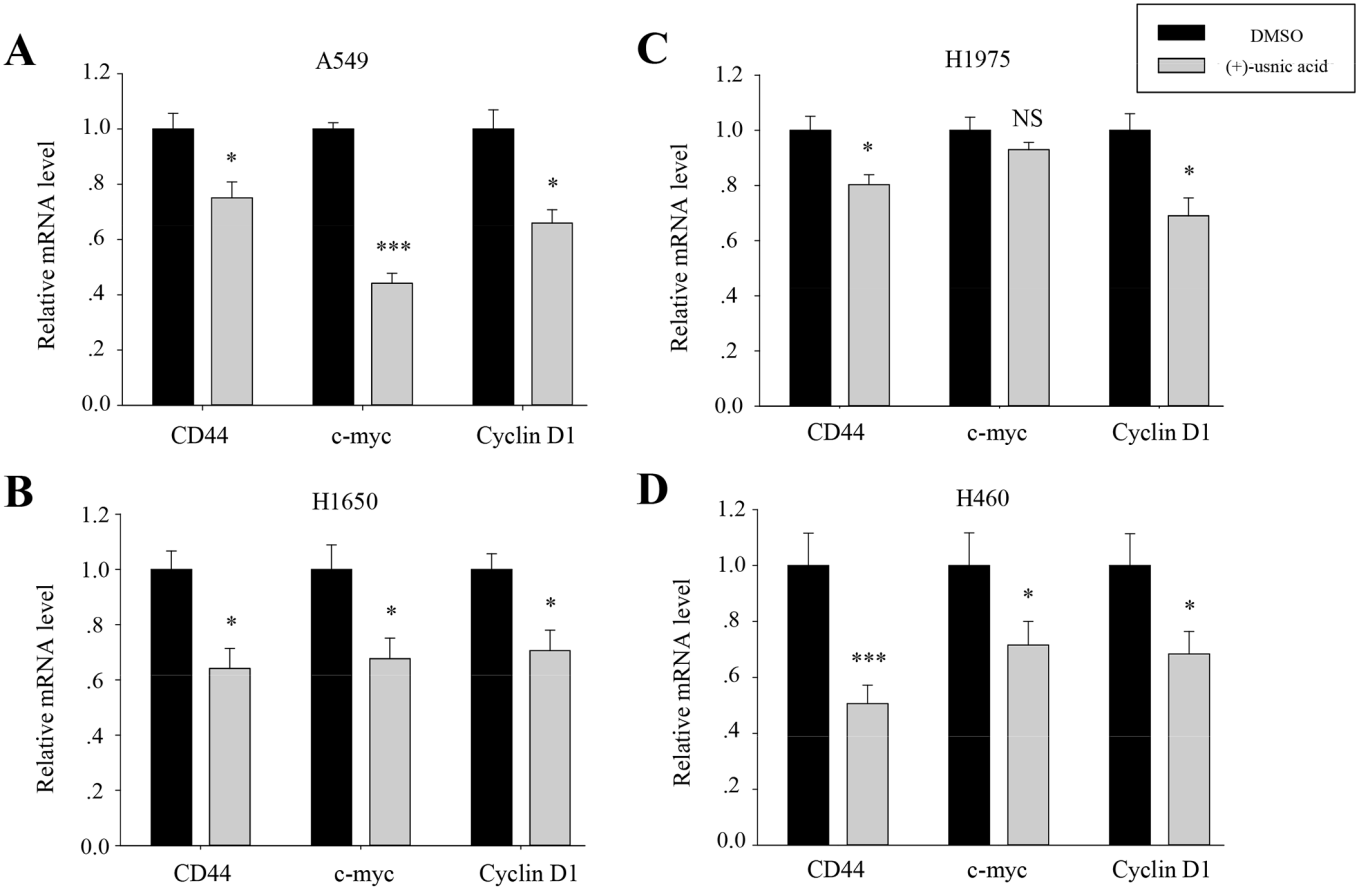
(+)-Usnic acid decreases mRNA level of downstream target genes of β-catenin/LEF and c-jun/AP-1. (A-D) Quantitative analysis of the mRNA level of CD44, c-myc, and Cyclin D1 in A549 (A), H1650 (B), H1975 (C), and H460 (D) cells treated with 5 μM of (+)-usnic acid. Data represent mean ± S.E.M. (standard error of the mean), n = 3. *p<0.05; ***p<0.001; NS, no significant difference when compared to the 0.01% DMSO-treated group in each cell line.

To investigate underlying mechanisms for the inhibitory activity of (+)-usnic acid, we performed TOPFLASH and AP-1 reporter assays to assess whether (+)-usnic acid can modulate β-catenin-mediated and/or KITENIN-mediated signaling activity. As shown in Fig 4A, (+)-usnic acid significantly decreased TOPFLASH activity by 18% at 1 μM and 37% at 10 μM. As for AP-1 activity, dose-dependent decreases were observed from 0.5 μM, and the decrease was significant, as much as 50% at 10 μM. Moreover, (+)-usnic acid also significantly decreased EGF-activated KITENIN-mediated AP-1 activity by 32% at 1 μM and 41% at 10 μM (Fig 4B). To check whether the level of downstream target genes of β-catenin/LEF and c-jun/AP-1 were affected by (+)-usnic acid treatment, quantitative real-time PCR analysis was performed. As shown in Fig 5, relative expression levels of CD44, cyclin D1, and c-myc were significantly decreased by (+)-usnic acid treatment in lung cancer cells to different extents (Fig 5A–5D). These results suggest that (+)-usnic acid shows inhibitory activity against cell motility through the modulation of β-catenin-mediated and KITENIN-mediated signaling activity in lung cancer cells.

### (+)-Usnic acid decreases GTP-Rac1 and -RhoA level

**Fig 6:**
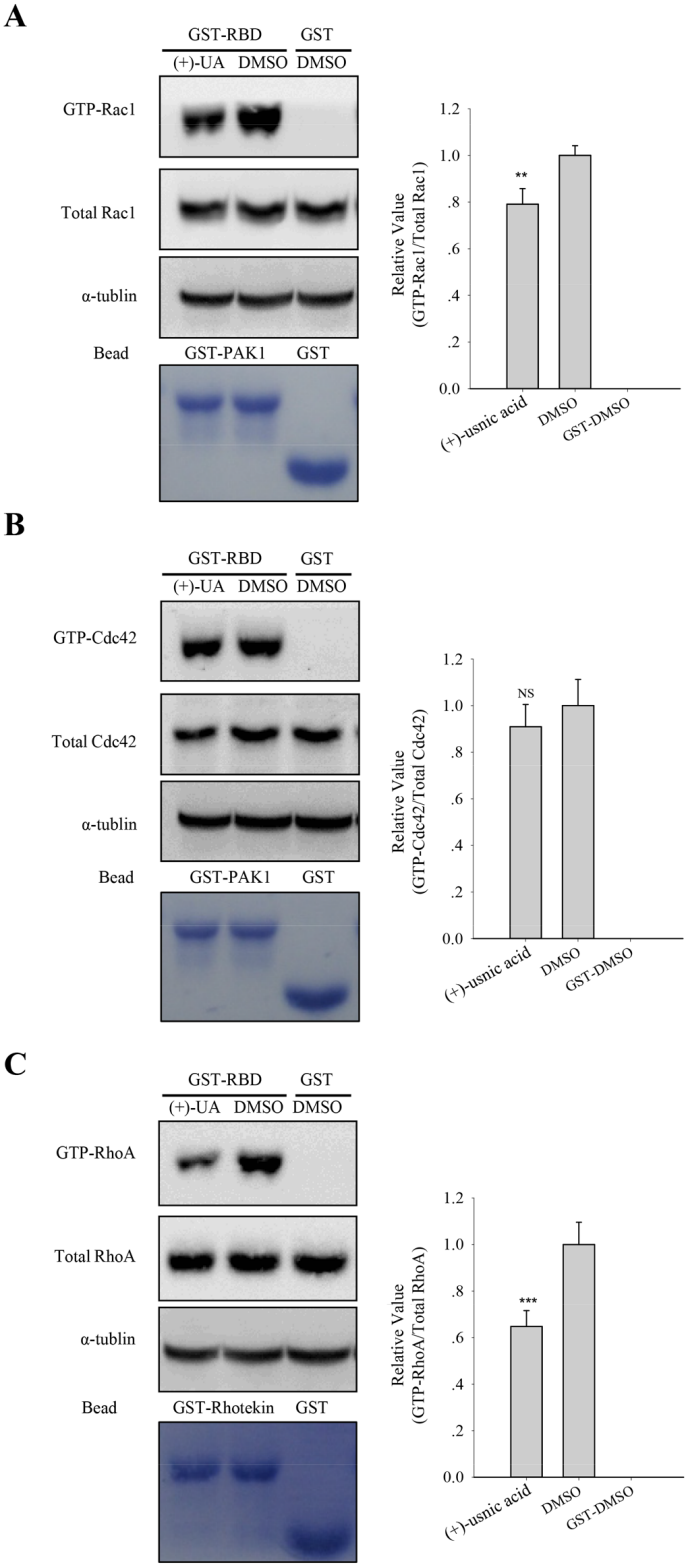
Regulation of RhoGTPases activity by (+)-usnic acid. (A-C) The levels of GTP-bound Rac1, Cdc42 and RhoA were measured in A549 cells treated with 5 μM of (+)-usnic acid. GTP-Rac1 and -Cdc42 were measured using GST-PBD, and GTP-RhoA was measured using GST-RBD. The total amounts of RhoA, Rac1, and Cdc42 were also shown. The relative activities of Rac1 (A), Cdc42 (B), and RhoA (C) were determined as described in Materials and Methods. The data represent the mean ± SEM (standard error of the mean), n = 3. **p<0.01; ***p<0.001; NS, no significant difference compared to 0.01% DMSO-treated A549 cells.

The activities of Rac1 and Cdc42 are involved in mesenchymal mode of migration [21, 22]. To determine whether (+)-usnic acid can affect the activities of these proteins in A549 cells, GST pull-down assays were performed using GST-PBD (p21-binding domain). As shown in Fig 6, (+)-usnic acid treatment significantly decreased the level of GTP-Rac1 by 22% compared to vehicle-treated cells (Fig 6A). However, no significant change in the level of GTP-Cdc42 was observed by (+)-usnic acid treatment (Fig 6B). RhoA promotes junctional formation, apical constriction, and reduces adhesion and cell spreading [23, 24]. To determine whether (+)-usnic acid can affect the activity of RhoA in A549 cells, GST pull-down assays were performed using GST-RBD (Rho-binding domain). As shown in Fig 6C, (+)-usnic acid treatment significantly decreased the level of GTP-RhoA by 40% compared to vehicle-treated cells. Taken together, these results suggest that (+)-usnic acid inhibits cell motility through the regulation of Rho GTPases.

### (+)-Usnic acid shows additive inhibitory activity with cetuximab

**Fig 7:**
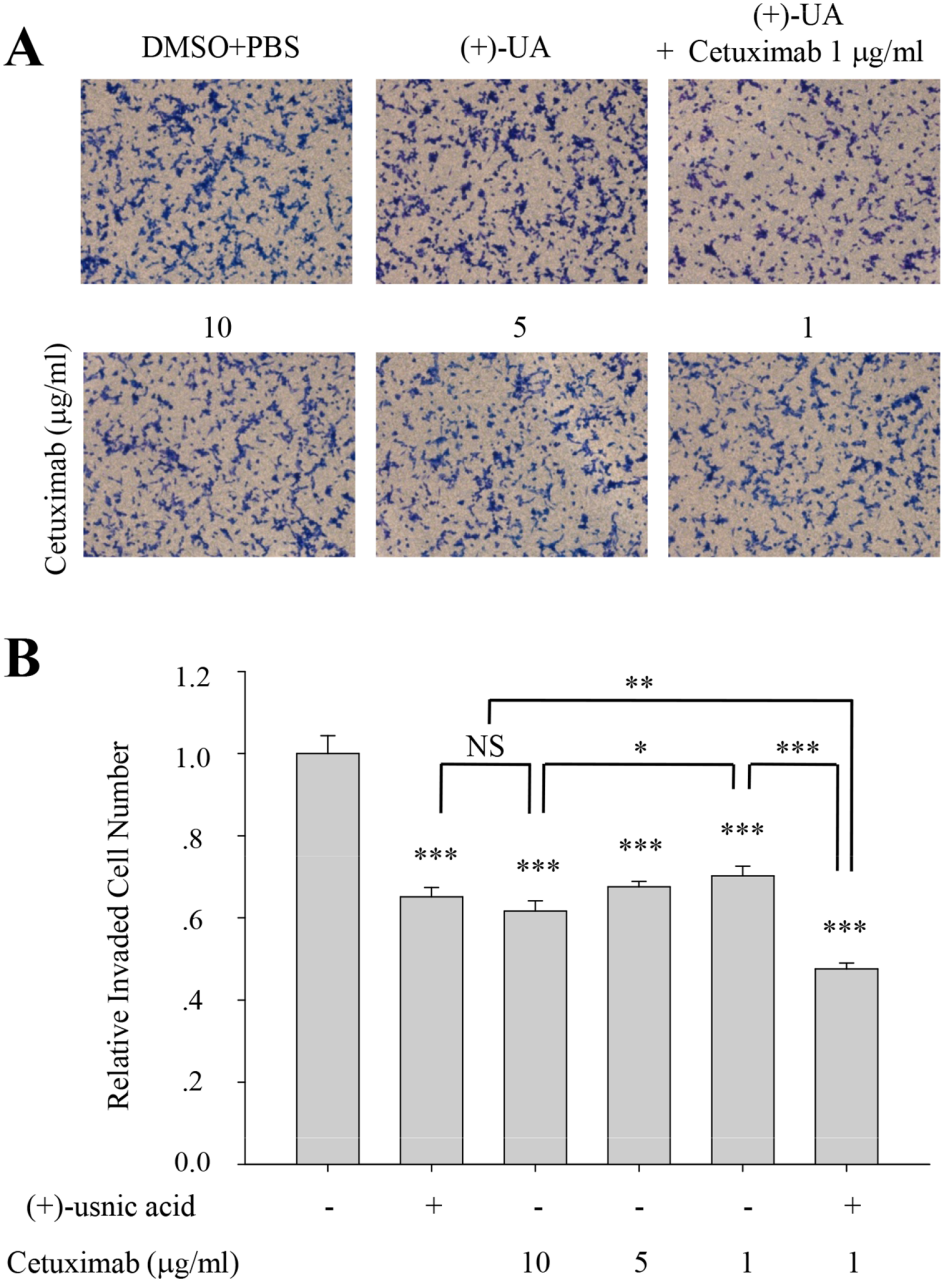
(+)-Usnic acid shows additive inhibitory activity with cetuximab. (A-B) Invasion assay of A549 cells treated with 5 μM of (+)-usnic acid and/or various concentration of cetuximab (A), and quantitative analysis of invaded cell numbers in each group (B). Representative images are shown from three independent experiments, n = 3. Data represent mean ± S.E.M. (standard error of the mean). *p<0.05; **p<0.01; ***p<0.001; NS, no significant difference between indicated group.

Cetuximab (Erbitux, C225), monoclonal antibody to epidermal growth factor receptor (EGFR) is used as one of anti-EGFR agents for the treatment of metastatic colon and lung cancer. To examine whether (+)-usnic acid has therapeutic relevancy with cetuximab, invasion assay was performed with various concentration of cetuximab and/or (+)-usnic acid. As shown in Fig 7, inhibitory activity of cetuximab was ~30% at 1 μg/ml and ~40% at 10 μg/ml on A549 cells, and treatment of 5 μM (+)-usnic acid showed similar inhibitory activity with 10 μg/ml of cetuximab (~40% inhibition) on these cells. Interestingly, higher inhibitory activity for cell invasion was observed when the cells were treated with 1 μg/ml of cetuximab together with 5 μM of (+)-usnic acid (Fig 7). These results suggest that (+)-usnic acid not only can inhibit lung cancer cell motility by alone but also can potentiate the therapeutic activity of cetuximab. As usnic acid decreased KITENIN-mediated AP-1 activity (Figs 4 and 5) and KITENIN/ErbB4-mediated downstream signal of EGF plays one of the molecular basis for conferring resistance to anti-EGFR agents [25], these results suggest that usnic acid may have potential beneficial activity in overcoming the limited clinical efficacy of anti-EGFR therapy.

## Discussion

Cancer cell acquires biological capabilities including resisting cell death, sustaining proliferative signaling, evading growth suppressors, activating invasion and metastasis, and so forth in developing from early to late stages [26, 27], and targeting either of these acquirements can be grouped as ‘anticancer’. In this regard, our observations that (+)-usnic acid inhibits migration and invasion ability in lung cancer cells are novel in anticancer activity of (+)-usnic acid. In addition, our results demonstrated that (+)-usnic acid have specific mechanisms of action for their anticancer activity, and these are quite different from those of previous literature showing cytotoxicity, one of mechanism of actions for the anticancer activity of (+)-usnic acid in various cancer cells.

Hepatotoxicity of usnic acid may restrict their potential medicinal use in cancer therapeutics [28]. However, most of hepatotoxicity in human was observed when high dose of usnic acid was orally administrated as a dietary supplement for the purpose of weight loss [29–31]. In cancer therapeutics, hepatotoxicity can be avoided by adjusting dosage, formulation and/or route of medication. For example, da Silva Santos et al. showed that nano-encapsulation of usnic acid enable to maintain and improve antitumor activity and considerably reduce the hepatotoxicity [32]. Furthermore, it has been shown that supplementation of anti-oxidant, i.e. vitamin E, together with usnic acid could greatly reduce the usnic acid-induced hepatotoxicity in primary cultured mouse hepatocytes [33]. It should also be noted that some of the papers showing hepatotoxicity were carried on HepG2 cells, originated from hepatocellular carcinoma tissue of 15 years male adolescent [34, 35]. In our previous paper [9], we demonstrated that usnic acid somehow has selective cytotoxicity in cancer cells when compared to normal cells. In alternative point of views, severe cytotoxicity of usnic acid on HepG2 cells reflects selective and specific anticancer activity of usnic acid on liver cancer in tested concentrations.

In our previous report, it was shown that the cytotoxicity of usnic acid is specific to cancer cells such as HT29 (colorectal cancer cells; IC_50_ = 95.2 ± 0.85 μM), AGS (gastric cancer cell; IC_50_ = 15.01 ± 0.52 μM), A549 (lung cancer cell; IC_50_ = 65.3 ± 0.65 μM), and CWR22Rv-1 (prostate cancer cells; IC_50_ = 24.1 ± 0.63 μM), while non-cancer cells such as MDCK (Mardin-Darby canine kidney; IC_50_ = 133.04 ± 3.5 μM), RIE (rat intestinal epithelial cells; IC_50_ = 126 ± 4.25 μM), NIH 3T3 (mouse embryonic fibroblast; IC_50_ = 164.2 ± 3.7 μM), and HaCaT (human keratinocyte; IC_50_ = 185.7 ± 4.8 μM) cells, were not severely damaged [9]. Given that the average circulating blood volume for mice is 72 mL/kg [36] and the molecular weight of usnic acid is 344, LD_50_ value of usnic acid (mouse-oral; 838 mg/kg) in MSDS sheet of usnic acid can be calculated to 33.8 mM. As inhibitory activity of usnic acid in inhibiting lung cancer cell motility is observed at concentration of 5 μM, our results indicated that usnic acid would be used for anti-metastasis agent with little toxicity at working concentrations.

Usnic acid has a low degree of aqueous solubility, which is an obstacle in the drug development as it is likely to result in poor bioavailability. To avoid this problem, various approaches can be made for the enhancement of solubility with its own compound by particle size reduction, crystal engineering, salt formation, solid dispersion, use of surfactant, complexation, and so forth [37]. The selection of the enhancement method should be determined to each drug in required dosage form characteristics. For usnic acid, recent article reported that potassium salt of usnic acid (potassium usnate) has 100% solubility without losing its biological activity at 10 μg/ml (approximately 26 μM) [38]. Together with the facts that (+) and (-) enantiomeric forms of usnic acid showed moderate to strong biological activities [39, 40], further study is required to evaluate the potential medicinal usage of usnic acid in anticancer therapy in various cancers.

## Supporting Information

S1 File LC-MS Analysis (Figure A in S1 File) and Optical activity Analysis (Figure B in S1 File) of samples used in this study. (PDF)

## Abbreviations

AP-1: activator protein-1
EGF: epidermal growth factor
DMSO: dimethylsulfoxide
HPLC: high performance liquid chromatography
KITENIN: KAI1 COOH-terminal interacting tetraspanin
LEF: lymphoid enhancing factor
PBD: p21-binding domain
PCR: polymerase chain reaction
RBD: Rho-binding domain
