# Supplementary material for: Inhibitory Activity of (+)-Usnic Acid against Non-Small Cell Lung Cancer Cell Motility
Source: PLoS One. 2016 Jan 11;11(1):e0146575. doi: 10.1371/journal.pone.0146575 (PMC4708991; doi:10.1371/journal.pone.0146575)
Supplement: S1 File — (PDF) [file pone.0146575.s001.pdf]

# Supplementary Material

**Figure A.** LC-MS Analysis of samples used in this study

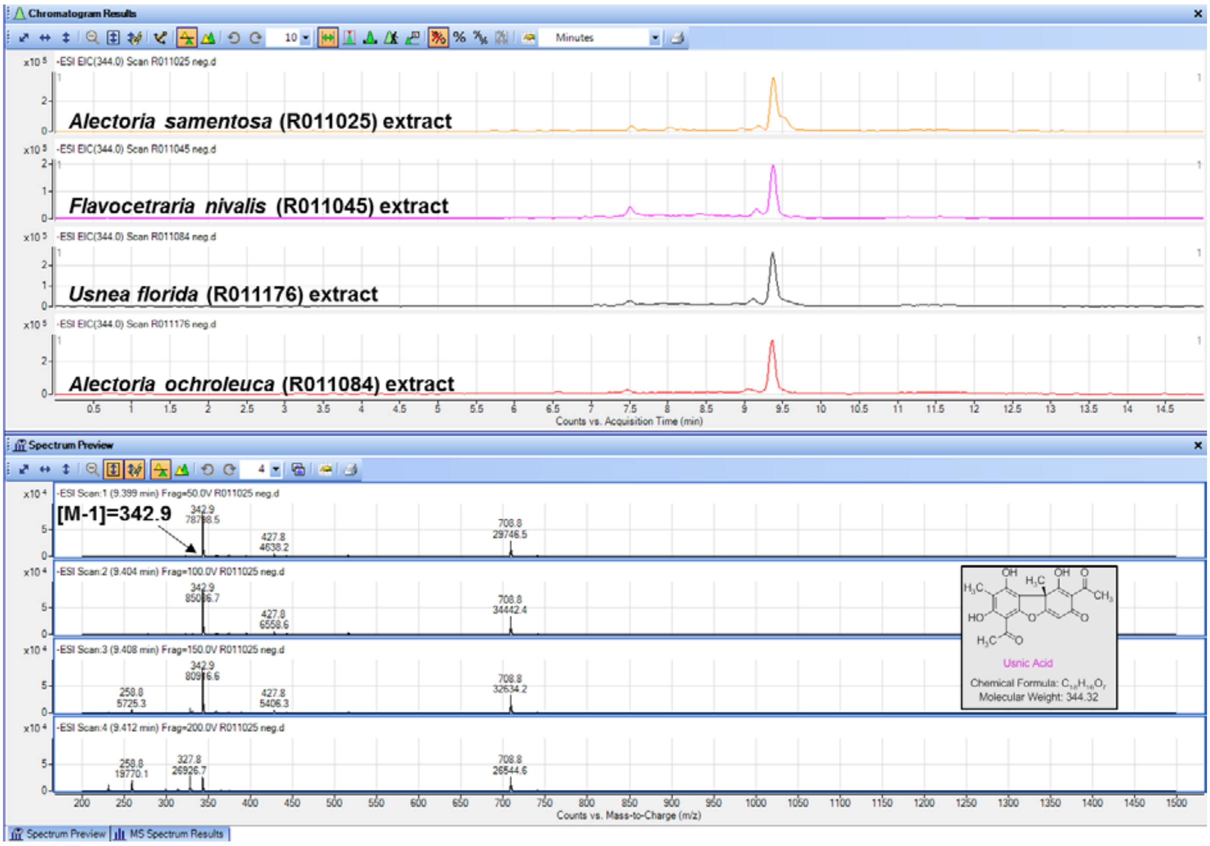

**Figure B.** Optical activity Analysis of samples used in this study.

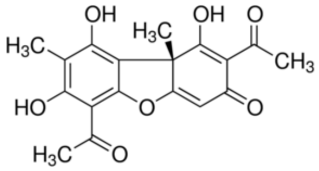


Reference data for optical activity of (+)-Usnic acid
**[α]25/D = +488°**, c = 0.7% in chloroform

| Collection No. | Samples | Optical activity | |
|---|---|---|---|
| | | Experimental Results | Reported usnic acid status in lichen species |
| - | (+)-Usnic acid (Sigma-Aldrich) | **[α]24.6 = +467° (c 1.7, $CHCl_3$)** | + |
| R011025 | *Alectoria samentosa* extract | **[α]25.0 = -345° (c 0.65, $CHCl_3$)** | - |
| R011045 | *Flavocetraria nivalis* extract | **[α]24.9 = -232° (c 1.42, $CHCl_3$)** | ± |
| R011176 | *Usnea florida* extract | **[α]24.9 = +107° (c 1.25, $CHCl_3$)** | + |
| R011084 | *Alectoria ochroleuca* extract | **[α]24.9 = -438° (c 1.25, $CHCl_3$)** | - |
